# Editorial: Artificial Intelligence (AI) Optimized Systems Modeling for the Deeper Understanding of Human Cancers

**DOI:** 10.3389/fbioe.2021.756314

**Published:** 2021-10-11

**Authors:** Zhiwei Ji, Shu Tao, Bing Wang

**Affiliations:** ^1^ College of Artificial Intelligence, Nanjing Agricultural University, Nanjing, China; ^2^ University of Texas Health Science Center at Houston, Houston, TX, United States; ^3^ UCLA Jonsson Comprehensive Cancer Center, Los Angeles, CA, United States; ^4^ Anhui University of Technology, Ma’anshan, China

**Keywords:** multi-scale modeling, multi-omics data, network modeling, single-cell RNA-seq analysis, big clinical data analysis

Cancer research in the field of Computational Systems Biology attempts to address questions that will advance current knowledge in the mechanisms of cancer progression or treatment resistance. By analyzing multi-omics data and developing a predictive mathematical and/or computational model of an unknown biological system, we can systematically understand 1) the mechanisms that tie altered gene expression and downstream molecular mechanisms to functional cancer phenotypes (Colaprico et al., 2020; Menyhárt and Győrffy, 2021); 2) and/or the mechanisms that tie tumor morphology to functional cancer phenotypes (Koutsogiannouli et al., 2013; Suhail et al., 2019); 3) and/or the mechanisms that tie treatment sequence and combination to evolving functional cancer phenotypes (Yalcin et al., 2020). Currently, systems biology still faces some challenges, including model calibration, model validation and generalization, computational efficiency, and the feasibility of clinical transition (Ching et al., 2018). Recent developments in artificial intelligence technologies, e.g., deep learning (DL), allow us to model the hierarchical structure of real biological systems, efficiently converting gene-level data to pathway-level information with an ultimate impact on cell phenotype (Gazestani and Lewis, 2019). Furthermore, such computational models could require fewer training samples, are more generalizable across diverse biological contexts, and can make predictions that are more consistent with the current understanding on the inner-workings of biological systems (Brodland, 2015).

This special issue entitled “*Artificial Intelligence (AI) Optimized Systems Modeling for the Deeper Understanding of Human Cancers*” in *Frontiers in Bioengineering and Biotechnology*, and *Frontiers in Genetics* aims to provide an international forum for:

1) bringing together the greatest research efforts in cancer-specific molecular/network signature identification by integrating multi-omics/multi-level data;
2) exploring future-generation interesting and practical biomedical applications in AI, machine learning, big data sciences, knowledge-based system, *etc.*, to provide novel ideas and solutions in mathematical modeling for tumor growth, drug resistance, and targeting effect prediction;
3) addressing the real-world challenges in the fields of AI-based patient diagnosis or disease progression prediction by utilizing modern machine learning or statistical strategies, and produce a more reliable and promising application environment to develop those technologies.

Submission for this special issue started from May 2020 and closed in Oct 2020. In nearly one and half years, we received in total 36 paper submissions. All submitted manuscripts had gone through at least two rounds of revision with reviewers in the related fields, including bioinformatics, computational biology, machine learning, and clinical study, *etc.* The final acceptance rate is 50% with 18 accepted papers in this special issue. The summaries of these papers are outlined below.

1) New bioinformatic approaches to Identify key molecular/network signatures for precision diagnosis or treatment of cancers

In the article entitled “*Identification of signatures of prognosis prediction for melanoma using a hypoxia score*” by Shou et al. The authors developed a computational method to identify the gene signatures of melanoma in hypoxic condition for prognosis prediction.

In the article entitled “*Identifying hypoxia characteristics to stratify prognosis and assess the tumor immune microenvironment in renal cell carcinoma*” by Zhang et al. The authors established a hypoxia-related risk model to predict the prognosis of patients.

In the article entitled “*Prediction of Radiosensitivity in Head and Neck Squamous Cell Carcinoma Based on Multiple Omics Data*” by Liu et al. The authors identified 12-gene signature based on multiple omics data achieved the best ability for predicting radiosensitivity in Head and Neck Squamous Cell.

In the article entitled “*An Effective Graph Clustering Method to Identify Cancer Driver Modules*” by Zhang et al. The authors proposed a graph clustering method, called “MCLCluster”, to identity cancer driver modules that drive cancer progression.

In the article entitled “*Exploring the differential expression and prognostic significance of the COL11A1 gene in human colorectal carcinoma: an integrated bioinformatics approach*” by Patra et al. The authors developed an integrated bioinformatics approach to reveal the COL11A1 gene as a prognostic biomarker in colorectal carcinoma.

In the article entitled “*MicroRNA-126 Modulates Palmitate-induced Migration in HUVECs by Downregulating Myosin Light Chain Kinase via the ERK/MAPK Pathway*” by Zhu et al. The authors evaluated the effects of miR-126 on the cell migration and uncovered the underlying mechanism in HUVECs treated with palmitate.

In the article entitled “*Integrated analysis of DEAD‐box helicase 56: a potential oncogene in osteosarcoma*” by Zhu et al. The authors set up a novel integrated analysis protocol and found that DDX56 is a potential therapeutic target for the treatment of osteosarcoma.

In the article entitled “*A machine learning approach for tracing tumor original sites with gene expression profiles*” by Liang et al. The authors developed a machine learning approach by integrating random forest and Naive Bayesian, to predict the primary origin sites of tumors.

In the article entitled “*A deep learning framework to predict tumor tissue-of-origin based on copy number variation*” by Liang et al. The authors proposed a deep learning framework composed of an autoencoder (AE) and a convolution neural network (CNN) to predict the primary origin sites of tumors.

In the article entitled “*TOOme: a novel computational framework to infer cancer tissue-of-origin by integrating both gene mutation and expression*” by He et al. The authors integrated somatic mutation and gene expressions t infer the primary original sites of tumor and obtained a great accuracy.

2) New studies of clinical informatics for speeding up the development of cancer diagnosis

In the article entitled “*Diagnosis of cervical cancer with parametrial invasion on whole-tumor dynamic contrast-enhanced magnetic resonance imaging combined with whole-lesion texture analysis based on T2-weighted images*” by Li et al. The authors integrated DCE-MRI images and texture analysis for diagnosis cervical cancer.

In the article entitled “*Predictive value of the texture analysis of enhanced computed tomographic images for preoperative pancreatic carcinoma differentiation*” by Zhang et al. The authors extracted 396 features from patient CT images and selected the optimal feature subset to predict the pathological degree of differentiation of pancreatic carcinoma.

In the article entitled “*RA-UNet: A hybrid deep attention-aware network to extract liver and tumor in CT scans*” by Jin et al. The authors developed a 3D network model, RA-UNet, to precisely extract the liver region and segment tumors from the liver. Testing on public datasets show that the proposed architecture obtains competitive results.

In the article entitled “*Classification of Infected Necrotizing Pancreatitis for Surgery within or beyond Four Weeks Using Machine Learning*” by Lan et al. The authors applied machine learning models to predict the optimal timing of surgical intervention and identified the key factors associated with surgical timing for infected necrotizing pancreatitis.

In the article entitled “*Prediction of Proximal Junctional Kyphosis after Posterior Scoliosis Surgery with Machine Learning in the Lenke 5 Adolescent Idiopathic Scoliosis Patient*” by Peng et al. The authors developed a machine learning model for proximal junctional kyphosis (PJK) prognostication in Lenke 5 adolescent idiopathic scoliosis (AIS) patients undergoing long posterior instrumentation and fusion surgery.

In the article entitled “*A New Method Based on CEEMD Combined with Iterative Feature Reduction for Aided Diagnosis of Epileptic EEG*” by Peng et al. The authors proposed a computational method based on complementary ensemble empirical mode decomposition (CEEMD) combined with iterative feature reduction for aided diagnosis of epileptic EEG.

3) New strategies for optimizing the data preprocessing and quality control

In the article entitled “*Assessing the impact of data preprocessing on analyzing next generation sequencing data*” by He et al. The authors compared commonly used data preprocessing software and found differences in the detection of hotspot mutations and HLA typing. They also explained the impact of data preprocessing steps on downstream data analysis results.

In the article entitled “*RF-PCA: A New Solution for Rapid Identification of Breast Cancer Categorical Data Based on Attribute Selection and Feature Extraction*” by Bian et al. The authors developed a hybrid model RF-PCA, which significantly reduce the time required for the classification, but also improved the accuracy.

The guest editors would like to thank all authors submitting their valuable works to this special section of Frontiers in Bioengineering and Biotechnology, Frontiers in Genetics, as well as all peer-reviewers for their great effort reviewing the submitted articles, providing constructive comments and suggestions and assisting the editors reaching the final decision. Special thanks will be sent to the editor-in-chief (EIC), Ranieri Cancedda and José AG Agúndez, for their precious time and valuable instructions that help us prepare and finalize this special issue.
